# A comprehensive overview of tolerogenic vaccine adjuvants and their modes of action

**DOI:** 10.3389/fimmu.2024.1494499

**Published:** 2024-12-20

**Authors:** Sabine Arve-Butler, Cody Deumont Moorman

**Affiliations:** ^1^ Amgen R&D Postdoctoral Fellows Program, Amgen Inc, South San Francisco, CA, United States; ^2^ Amgen Research, Amgen Inc., South San Francisco, CA, United States

**Keywords:** tolerogenic adjuvant, tolerogenic vaccine, autoimmune disease, immune tolerance, regulatory T cells, tolerogenic dendritic cells, immunoregulation, adjuvant

## Abstract

Tolerogenic vaccines represent a therapeutic approach to induce antigen-specific immune tolerance to disease-relevant antigens. As general immunosuppression comes with significant side effects, including heightened risk of infections and reduced anti-tumor immunity, antigen-specific tolerance by vaccination would be game changing in the treatment of immunological conditions such as autoimmunity, anti-drug antibody responses, transplantation rejection, and hypersensitivity. Tolerogenic vaccines induce antigen-specific tolerance by promoting tolerogenic antigen presenting cells, regulatory T cells, and regulatory B cells, or by suppressing or depleting antigen-specific pathogenic T and B cells. The design of tolerogenic vaccines vary greatly, but they all deliver a disease-relevant antigen with or without a tolerogenic adjuvant. Tolerogenic adjuvants are molecules which mediate anti-inflammatory or immunoregulatory effects and enhance vaccine efficacy by modulating the immune environment to favor a tolerogenic immune response to the vaccine antigen. Tolerogenic adjuvants act through several mechanisms, including immunosuppression, modulation of cytokine signaling, vitamin signaling, and modulation of immunological synapse signaling. This review seeks to provide a comprehensive examination of tolerogenic adjuvants currently utilized in tolerogenic vaccines, describing their mechanism of action and examples of their use in human clinical trials and animal models of disease.

## 1 Introduction

Immunological tolerance is a state of unresponsiveness or an anti-inflammatory response that promotes immune homeostasis and prevents detrimental immune reactions directed toward self-antigens and tissues (1). Tolerogenic vaccines aim to induce antigen-specific tolerance in conditions where tolerance has failed, or where there is an aberrant inflammatory response toward antigens not associated with danger. Tolerogenic vaccines differ from general immunosuppression or immunomodulation in that they aim to induce specific immune tolerance to the disease-relevant antigens, thereby suppressing the autoimmune response without affecting the immune system as a whole (2, 3). Autoimmune disease, transplantation rejection, anti-drug antibody responses, and hypersensitivity all represent conditions where tolerogenic vaccines are promising new therapeutic regimens (2, 3).

Tolerogenic vaccines can induce antigen-specific tolerance via effects on key players in peripheral tolerance. Mechanisms of immune tolerance are reviewed extensively elsewhere (1, 4, 5). In brief, master regulators of peripheral tolerance toward specific antigens are regulatory T cells (Tregs) and tolerogenic dendritic cells (tolDCs) (1, 3–6). Tregs exert their immunoregulatory effects via anti-inflammatory cytokines, direct suppression of conventional T cell (Tcon) proliferation, and by modulating DC maturation and function. TolDCs act by promoting Treg development, suppressing effector T cell responses, and by inducing antigen-specific T cell anergy. Many other cell types can contribute to immunological tolerance, of note are B cells producing anti-inflammatory cytokines (7) and type 1 regulatory T cells (Tr1 cells) (8).

**Figure 1:**
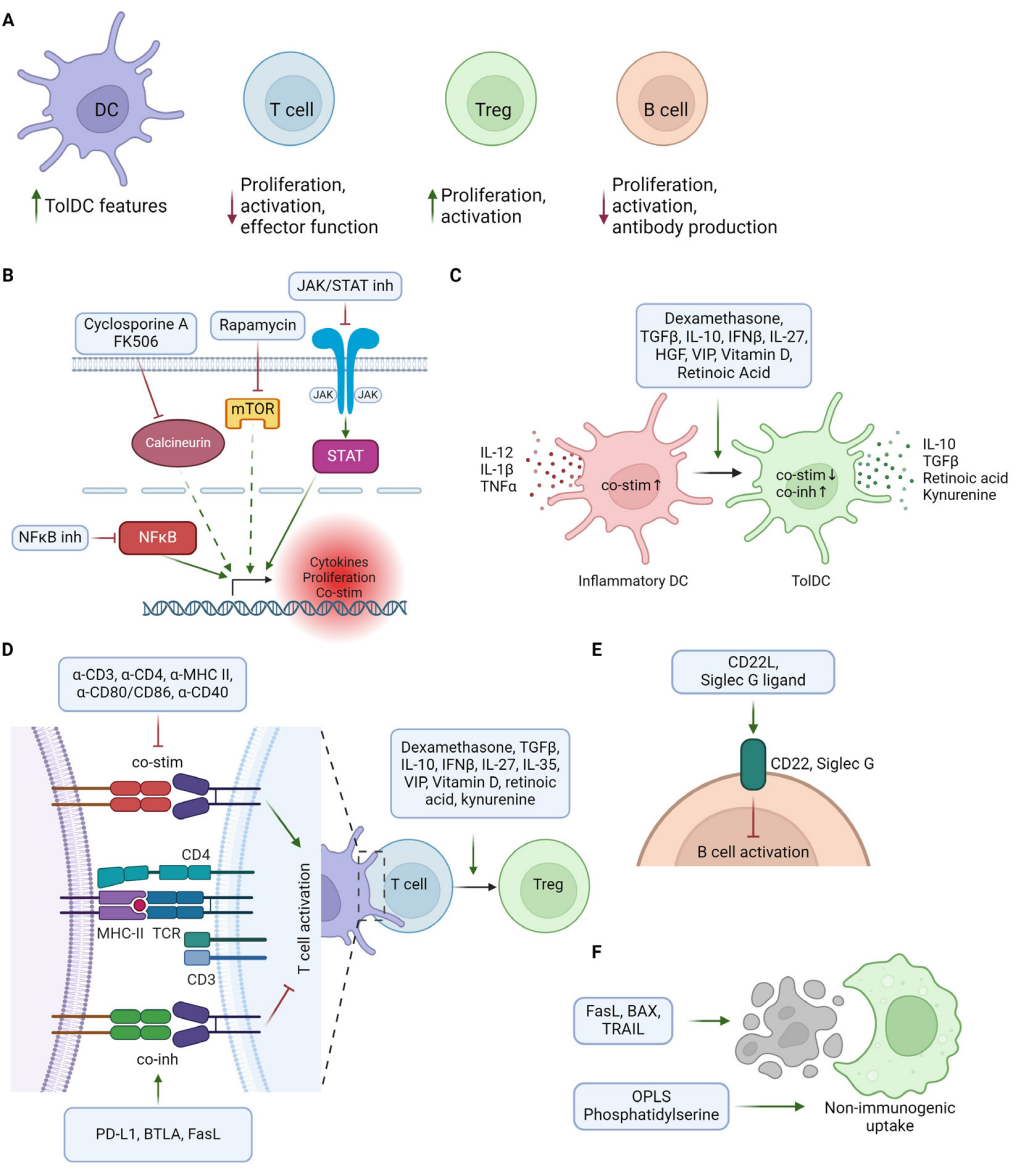
Schematic overview of tolerogenic adjuvants mechanisms of action. **(A)** Overview of main tolerogenic effects of adjuvants on different immune cell types, including suppression of immune activation and promotion of tolerogenic/immunoregulatory cells. **(B)** Immunoregulation through direct or indirect suppression of pro-inflammatory gene expression. **(C)** Adjuvants supporting differentiation from inflammatory DCs (red) to tolerogenic DCs (green). TolDCs are characterized by lower levels of co-stimulatory and increased levels of co-inhibitory molecules, together with release of Treg-promoting cytokines and metabolites. **(D)** Regulation of T cell responses by modulation of the immunological synapse or Treg promoting factors. Tolerogenic adjuvants can suppress T cell activation by inhibiting co-stimulation and/or promoting co-inhibition in the interaction between T cells and antigen presenting cells. Treg differentiation can be enhanced both by APC-T cell interactions and by Treg promoting factors acting on T cells. **(E)** Suppression of B cell activation by activation of inhibitory receptors. **(F)** Promoting non-immunogenic uptake of tolerogenic vaccine by mimicking or inducing apoptotic cells. Created in BioRender.

**Figure 2:**
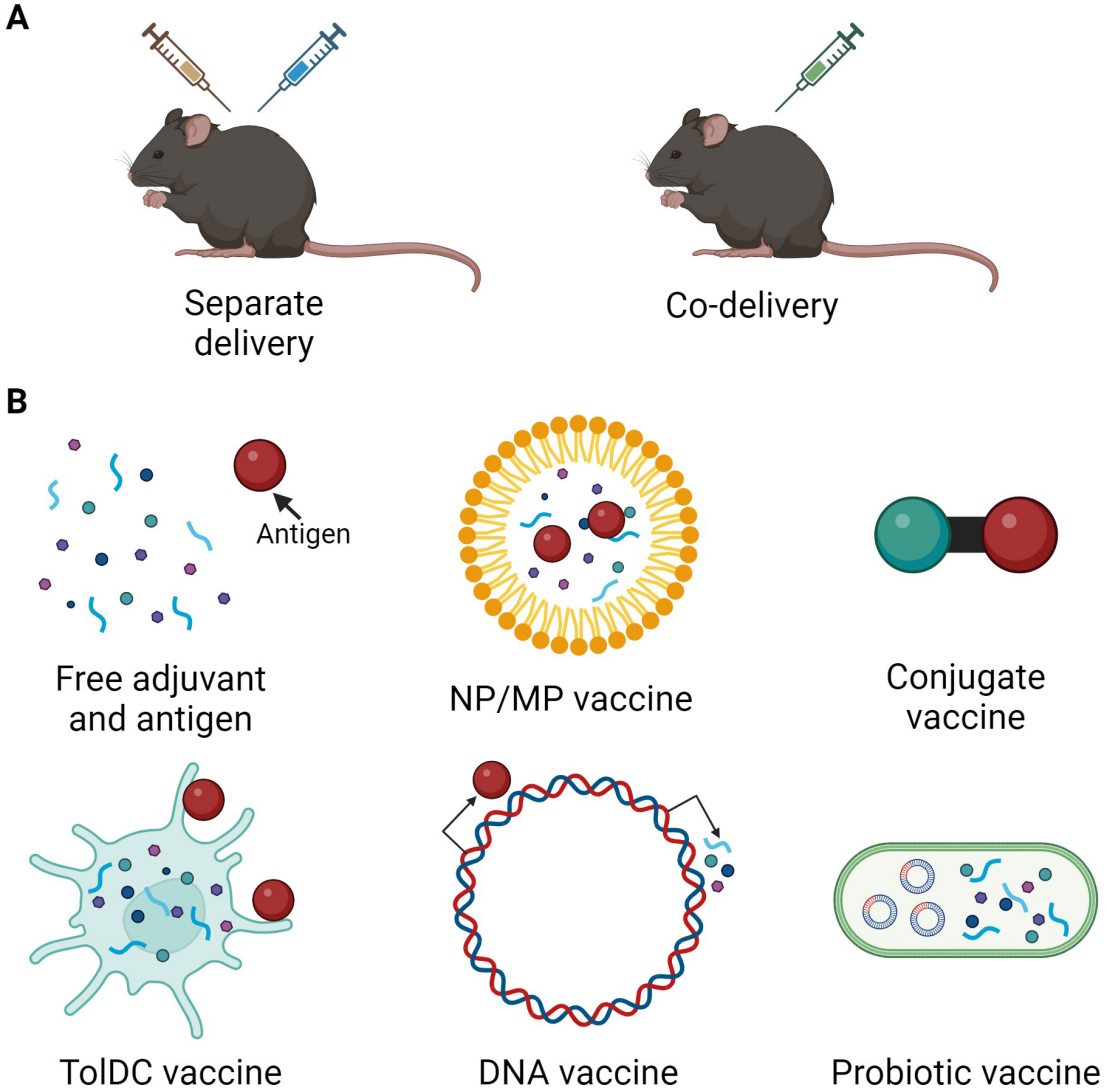
Overview of tolerogenic vaccine design and delivery. **(A)** Tolerogenic vaccine delivery types. **(B)** Different types of tolerogenic vaccines include administration of free adjuvant and antigen, incorporation of antigen and/or adjuvant in nanoparticles (NP) or microparticles (MP), conjugates or fusion molecules of antigen and adjuvant, antigen-loading of adjuvant-treated TolDCs, delivery of DNA vectors encoding antigen and/or adjuvant, and probiotic bacteria engineered to express antigen and/or adjuvant. Created in BioRender.

Adjuvants are molecules used to enhance the effect of pharmacological treatments (9), therefore tolerogenic adjuvants aim to increase anti-inflammatory responses and enhance vaccine efficacy. A schematic overview of the main mode(s) of action for tolerogenic adjuvants described in this review are depicted in **Figure 1**. Tolerogenic vaccines use adjuvants in different ways depending on vaccine type. Antigen and adjuvants can be administered either separately or co-delivered, where the co-delivery can range from simultaneous administration of free antigen and adjuvant to intricate delivery systems, fusion molecules, or DNA vectors (**Figure 2**). Tolerogenic adjuvants can also be used to differentiate tolDCs for cell transfer of antigen-loaded tolDCs (**Figure 2**).

The tolerogenic adjuvants described in this review are grouped in five categories based on their properties and mechanism of action: general immunosuppressive agents (**Table 1**), cytokines and neuropeptides (**Table 2**), vitamins and vitamin derivatives (**Table 2**), modulators of contact-dependent immune cell signaling (**Table 3**), and other (**Table 4**). In tolerogenic vaccines using a combination of adjuvants, tolerance can be mediated by multiple mechanisms (**Table 5**). Tolerogenic vaccines in human clinical trials are discussed for each category and summarized in **Table 6**.

**Table 1:** Immunosuppressive adjuvants.

Adjuvant	Antigen	Formulation	Administration	Disease Model	Major Results	Ref
Dexamethasone	PLP^139-151^	Conjugate Vaccine: Adj-Ag	Prophylactic: s.c.	EAE	↓ Disease Incidence ↓ IL-2+ Cells	(19)
Dexamethasone	MOG^35-55^	Conjugate Vaccine: Adj-Ag	Prophylactic: s.c.	EAE	↓ Disease Score ↓ T Cell Responses ↓ Ag-Th17 ↑ TolDC	(20)
Dexamethasone	HSP60^292–308^	Separate Delivery Vaccine: Adj and Ag	Prophylactic: Adj i.m. Ag s.c.	Atherosclerosis	↓ Disease Severity ↓ T Cell responses ↑ Ag-Tregs ↑ B-1	(23)
Dexamethasone	InsB^9-23^	Separate Delivery Vaccine: Adj and Ag	Prophylactic: foot pad injection	T1D	↓ Disease Incidence ↑ TolDC ↑ Tregs, IL-10 Tregs	(25)
Dexamethasone	MOG^35-55^	MP Vaccine: MPs containing Adj and Ag	Therapeutic: s.c.	EAE	↓ Disease Incidence ↓ IL-17, GM-CSF ↑ TolDC	(22)
Dexamethasone	Human proteoglycan	MP Vaccine: MPs containing Adj and Ag	Prophylactic: i.v. Therapeutic: i.v.	AIA	↓ Disease Incidence ↓ Disease Score ↓ Ag-antibodies ↑ Tr1 ↑ TolDC	(24)
Dexamethasone	B. tropicalis	TolDC Vaccine: BMDC treated with Adj, loaded with Ag	Prophylactic: i.p.	Allergy	↓ Cell Infiltration ↓ Eosinophils ↓ Ag-Antibodies, IgE ↓ IFN-γ, IL-4 ↑ Tregs	(28)
Dexamethasone	MOG^35-55^	NP Vaccine: NPs containing Ag-Adj conjugates	Therapeutic: i.v.	EAE	↓ Disease Score (with and without Dexamethasone) ↑ TolDC ↑ Tregs	(21)
Rapamycin	Amyloid β	NP Vaccine: NPs containing Adj and Ag	Therapeutic: i.v.	Alzheimer’s	↓ Cognitive Decline ↓ IFN-γ ↑ TGF-β, IL-10, Arg1 ↑ Tregs ↑ TolDC	(39)
Rapamycin	Citrullinated proteins	NP Vaccine: NPs containing Adj and Ag	Therapeutic: i.v Prophylactic: i.v.	CIA, AIA	↓ Disease Score ↓ IFN-γ, IL-17, TNF, IL-1β ↑ TGF-β, IL-10 ↑ Tregs	(38)
Rapamycin	HEL^46-61^	NP Vaccine: NPs containing Adj and Ag	Prophylactic: i.v.	Vitiligo	↓ Disease Score ↓ Ag-T Responses ↓ IL-6, IFN-γ ↑ IL-10 ↑ Tregs ↑ TolDC	(40)
Rapamycin	OVA	NP Vaccine: NPs containing Adj and Ag	Prophylactic: i.v.	Anaphylaxis, Allergy	↓ Disease Score ↓ Ag-Antibody ↓ Eosinophils, Neutrophils ↓ IL-4, IL-5 ↑ Tregs ↑ TGF-β	(41)
Rapamycin	P31	NP Vaccine: NPs containing Adj and Ag	Prophylactic: s.c.	T1D	↓ Disease Incidence ↑ Tregs	(43)
Rapamycin	PLP^139-151^ OVA FVIII	NP Vaccine: NPs containing Adj and Ag	Therapeutic: i.v. and s.c. Prophylactic: i.v. and s.c.	EAE, Anaphylaxis, ADA	↓ Disease Score ↓ Ag-Antibody ↓ Eosinophils ↑ Ag-Tregs	(44, 45)
Rapamycin	FVIII	NP Vaccine: NPs containing Adj and Ag	Therapeutic: i.v. Prophylactic: i.v	ADA	↓ Ag-Antibody	(46)
Rapamycin	Uricase Adalimumab (anti-TNFα)	Co-Delivery Vaccine: NPs containing Adj and free Ag	Prophylactic: s.c.	ADA	↓ Ag-Antibody ↓ Disease Score	(47)
Rapamycin	OVA^323-339^	Separate Delivery Vaccine: Adj and Ag	Prophylactic: i.p.	Graft rejection	↓ Graft Rejection ↑ Ag-Tregs	(51)
Rapamycin	FVIII	Separate Delivery Vaccine: Adj and Ag	Prophylactic: i.v.	ADA	↓ Ag-Antibody ↓ IL-6, IL-2, IL-4 ↑ Tregs ↑ CTLA-4 ↑ TGF-β	(52)
Rapamycin	OVA	NP Vaccine: NPs containing Adj and Ag	Prophylactic: s.c.	Allergy	↓ Ag-Antibody ↓ Eosinophils, Cell Infiltration ↓ Th17, Th2 ↑ Tregs ↑ IL-10+ T Cells	(42)
Rapamycin	AAV8	NP Vaccine: NPs containing Adj, separate Ag vector	Prophylactic: i.v.	ADA	↓ Ag-Antibody ↓ B and T Cell Activation	(48)
Cyclosporine A	GAD65^206-220^ GAD65^536-55^ InsB^9-23^ InsC^17-A1^	Co-Delivery Vaccine: Free Ag and Adj	Prophylactic: s.c.	T1D	↓ Disease Incidence ↓ T Cell Responses ↓ TNF-α+, IL-2+ Cells ↓ Th1 ↑ Ag-Tregs ↑ IL-10 ↑ TolDC	(56)
FK506	CII	TolDC Vaccine: MoDC treated with Adj, loaded with Ag	Therapeutic: i.p.	CIA	↓ Disease Score ↓ T Cell Responses ↓ Th17	(58)
FK506	MOG^35-55^	DNA Vaccine/Co-delivery Vaccine: Adj and separate DNA Ag construct	Prophylactic: i.m.	EAE	↓ Disease Score ↓ T Cell Infiltration ↓ Th17, ↓ IFN-γ ↑ Tregs ↑ IL-4	(57)
Kynurenine	GAD65	Co-Delivery Vaccine: Adj and Ag phage vaccine	Prophylactic: s.c.	T1D	↓ Disease Incidence ↓ Ag T Cell Responses ↓ DC Activation ↓ IFNγ, IL-2 ↑ Tregs ↑ IL-10, TGF-β	(70)
ITE	MOG^35-55^ PLP^139-151^ Proinsulin	NP Vaccine: NPs containing Adj and Ag	Therapeutic: i.p. Prophylactic: i.p.	EAE	↓ Disease Score ↓ Ag-Response ↓ IFN-γ, IL-17 ↓ Th17 ↑ TolDC, Tregs	(67–69)
Andrographolide	FVIII	TolDC Vaccine: BMDC treated with Adj, loaded with Ag	Prophylactic: i.v.	ADA	↓ Ag-Antibody ↓ IL-4, IFN-γ ↑ Tregs	(61)
A20	OVA	NP Vaccine: Adj and Ag	Therapeutic: nasal	Asthma	↓ Disease Score ↓ Ag- IgE ↓ IL-4, IL-5, IL-13 ↑ Foxp3	(63)
BAY 11-7082	mBSA	TolDC Vaccine: BMDC treated with Adj, loaded with Ag	Therapeutic: s.c.	AIA	↓ Disease Score ↓ Ag-Antibody	(60)
Tofacitinib	MOG^35-55^	TolDC Vaccine: BMDC treated with Adj, loaded with Ag	Prophylactic: i.v.	EAE	↓ Disease Score ↓ Ag-T Cell Responses ↓ Th17, Th1 ↑ Tregs	(72)
BD750	MOG^35-55^	TolDC Vaccine: BMDC treated with Adj, loaded with Ag	Prophylactic: i.v.	EAE	↓ Disease Score ↓ T Cell Responses ↓ Th17, Th1 ↓ IFN-γ, IL-17 ↑ Tregs ↑ IL-10	(73)
Rosiglitazone	CII	TolDC Vaccine: BMDC treated with Adj, loaded with Ag	Therapeutic: s.c.	CIA	↓ Disease Score ↓ Th1 ↑ Tregs	(75)
K313	MOG^35-55^	TolDC Vaccine: BMDC treated with Adj, loaded with Ag	Therapeutic: i.v.	EAE	↓ Disease Score ↓ Th17, Th1 ↑ Tregs	(77)
Iloprost	OVA^323-339^	TolDC Vaccine: BMDC treated with Adj, loaded with Ag	Prophylactic: intrathecally	Allergy	↓ Disease Score ↓ Infiltrating Cells ↓ Eosinophils ↓ IL-5, IL-4, IL-13, IFN-γ ↑ Tregs	(80)

AAV8, Adeno-associated virus 8; ADA, Antidrug antibody; Adj, Adjuvant; Ag, antigen; AIA, antigen-induced inflammatory arthritis; BMDC, bone marrow-derived dendritic cells; CD, cluster of differentiation; CIA, collagen-induced arthritis; CII, type II collagen; DC, dendritic cell; DNA, deoxyribonucleic acid; EAE, experimental autoimmune encephalomyelitis; FOXP3, forkhead box P3; FVIII, Factor VIII; GAD65, glutamic acid decarboxylase; HEL, Hen egg lysozyme; HSP60, Heat shock protein 60; IFN-γ, interferon-γ; i.m., intramuscular; ITE, 2-(1′H-indole-3′-carbonyl)-thiazole-4-carboxylic acid methyl ester; IL, interleukin; Ins, Insulin; i.p., intraperitoneal; i.v., intravenous(ly); mBSA, Methylated bovine serum albumin; MOG, myelin oligodendrocyte glycoprotein; MP, microparticle; NP, nanoparticle; OVA, ovalbumin; PBMCs, peripheral blood mononuclear cells; PLP, proteolipid protein; s.c., subcutaneous(ly); T1D, type 1 diabetes; Th; T helper; TGF-β, Transforming Growth Factor-β; TNF, tumor necrosis factor; Tr1, type 1 regulatory T cell; Treg, regulatory T cell; TolDC, tolerogenic dendritic cell.

↓, decrease; ↑, increase.

**Table 2:** Cytokine and vitamin adjuvants.

Adjuvant	Antigen	Formulation	Administration	Disease Model	Major Results	Ref
TGF-β	PLP	NP Vaccine: Adj and Ag	Therapeutic: i.v. Prophylactic: i.v.	EAE	↓ Disease Score ↓ T Cell Responses ↓ DC Activation ↓ IL-6, IL-12 ↑ Tregs ↑ TGF-β	(83)
TGF-β	MOG	DNA Vaccine: Adj and Ag constructs	Prophylactic: i.d.	EAE	↓ Disease Score ↓ T Cell Infiltration ↓ Th17, Th1 ↓ INF-γ, IL-17	(94)
TGF-β	FVIII	TolDC Vaccine: BMDCs treated with Adj, loaded with Ag	Prophalctic: i.v.	ADA	↓ Ag-Antibody ↓ IL-2 ↑ Tregs ↑ IL-10	(85)
TGF-β signaling agonist (T74)	CII	TolDC Vaccine: BMDC treated with Adj, loaded with Ag	Therapuetic: s.c.	CIA	↓ Disease Score ↓ INF-γ ↓ Th1, Th17 ↑ Tregs ↑ IL-10	(84)
IL-10	MBP^68-86^	DNA Vaccine: Plasmid encoding Adj and plasmid encoding Ag	Therapeutic: i.p. Prophylactic: i.p.	EAE	↓ Disease Score ↓ Ag-T Cell ↑ IL-10	(103)
IL-10	MOG^35-55^	NP Vaccine: Separate NPs with Adj and Ag	Prophylactic: s.c.	EAE	↓ Disease Score ↓ T Cell Infiltration ↓ IFN-γ, IL-17	(101)
IL-10	OVA	TolDC Vaccine: BMDCs retrovirally transduced with IL-10 construct, loaded with Ag	Prophylactic: i.t. Therapeutic: i.t.	Asthma	↓ Disease Score ↓ Cell Infiltration ↓ IFN-γ, IL-4 ↓ Autoantibodies ↑ Tregs	(98)
IL-10	GAD65^190-315^	DNA Vaccine: Adj and Ag construct	Prophylactic: i.m.	T1D	↓ Disease Incidence ↑ IL-10, IL-4 ↑ Th2 ↑ Ag-Tregs	(102)
IL-10	MOG^35-55^	TolDC Vaccine: BMDCs treated with adj, loaded with Ag	Prophylactic: i.v.	EAE	↓ Disease Score ↓ Ag-T Cell Responses	(100)
IL-10	InsB^4-29^ BDC2.5mi	TolDC Vaccine: BMDCs transduced with IL-10 construct, loaded with Ag	Prophylactic: i.p.	T1D	↓ Disease Incidence ↑ Tr1	(99)
IL-2	IRBP	Separate Delivery Vaccine: Free Ag and Adj	Prophylactic: Ag orally adj i.p.	EAU	↓ Disease Score ↑ TGF-β, IL-10, IL-4	(111)
IL-2	MBP^69-88^	Conjugate Vaccine: Adj-Ag fusion protein	Therapeutic: s.c. Prophylactic: s.c.	EAE	↓ Disease Score	(109)
IL-2/anti-IL-2 complex	OVA	Separate Delivery Vaccine: Free Ag and Adj	Prophylactic: i.v.	DTH	↓ Disease Score ↑ Tregs	(112)
IL-2/anti-IL-2 complex	BDC2.5mi	Separate Delivery Vaccine: Free Ag and Adj	Prophylactic: i.p.	T1D	↓ Disease Incidence ↑ Ag-Tregs ↑ Treg Function	(110)
IL-2/anti-IL-2 complex	FVIII	Separate Delivery Vaccine: Free Ag and Adj	Prophylactic: Adj i.p., Ag i.v.	ADA	↓ Ag-Antibodies ↓ Ag-T Cell Responses ↑ Tregs	(113)
GM-CSF	MOG^35-55^ PLP^139-151^ MBP^68-87^	Conjugate Vaccine: Adj-Ag fusion protein	Therapeutic: s.c. Prophylactic: s.c.	EAE	↓ Disease Incidence ↑ Ag-Tregs	(125–129)
GM-CSF	IRBP^161-180^	TolDC Vaccine: BMDCs treated with adj, loaded with Ag	Prophylactic: s.c.	EAU	↓ Disease Score ↓ IL-2, IFN-γ ↑ IL-4, IL-5	(130)
IFN-β	MOG^35-55^ PLP^178-191^	Co-Delivery Vaccine: Adj and Ag in Alum or Adj-Ag fusion protiens	Therapeutic: s.c. Prophylactic: s.c.	EAE	↓ Disease Score ↑ Ag Tregs	(120, 121)
IL-35	MOG^35-55^	TolDC Vaccine: Transformed DC line transduced with IL-35 construct, loaded with Ag	Prophylactic: i.v.	EAE	↓ Disease Score ↓ Th1 ↓ T Cell Responses	(133)
IL-35-Ig	HY peptide	TolDC Vaccine: Splenic DCs transduced with IL-35-Ig construct, loaded with Ag	Prophylactic: i.v.	DTH	↓ Disease Score ↑ CD39^+^ Tregs ↑ Arginase 1	(134)
IL-27	MOG^35-55^ PLP^178-191^	TolDC Vaccine: BMDCs treated with adj, loaded with Ag	Therapeutic: i.v. Prophylactic: i.v.	EAE	↓ Disease Score ↓ Ag-T Cell Responses ↓ IFN-γ, IL-17 ↑ Tregs ↑ IL-10, TGF-β	(136)
IL-4	GAD^65^	DNA Vaccine: Transgenic plant leaf expressing Ag + Adj	Prophylactic: oral	T1D	↓ Disease Incidence ↓ IFN-γ ↑ Regulatory Cells ↑ IL-4	(141)
IL-4	GAD^65^	DNA Vaccine: Ag-IgGFc, fusion and Adj constructs	Prophylactic: i.m.	T1D	↓ Disease Incidence ↓ IFN-γ ↑ IL-4, IL-5	(142)
IL-4	PLP^139-151^ MOG	DNA Vaccine: Adj and Ag construct	Therapeutic: i.m. Prophylactic: i.m.	EAE	↓ Disease Score ↓ Ag-T Cell Response ↓ IFN-γ ↑ IL-4, IL-10	(139)
IL-4	CII	DNA Vaccine: Adj + Ag construct	Prophylactic: i.m.	CIA	↓ Disease Score ↓ TNF, IFN-γ	(140)
HGF	MOG^35-55^	TolDC Vaccine: Primary DCs treated with Adj, loaded with Ag	Therapeutic: i.v.	EAE	↓ Disease Score ↓ Infiltrating Cells ↓ Th17, Th1 ↓ IFN-γ, IL-17 ↑ Tregs ↑ IL-10, TGF-β	(144)
VIP	CII MOG	TolDC Vaccine: BMDCs treated with adj, loaded with Ag	Therapeutic: i.v.	CIA EAE	↓ Disease Score ↓ Ag-response, Ag-antibodies ↓ IFN-γ ↑ Tr1 ↑ IL-10	(146)
TRAIL	MOG^35-55^	TolDC Vaccine: BMDC treated with Adj plasmid and Ag plasmid	Therapeutic: i.v.	EAE	↓ Disease Score ↓ Ag-Response ↓ Cell Infiltration ↑ Tregs	(148, 149)
BAFF-siRNA	CII	TolDC Vaccine: BMDC transduced with Adj construct, loaded with Ag	Therapeutic: i.v.	CIA	↓ Disease Score ↓ Ag-antibody ↓ Rorγt ↓ IL-17, IL-1β, IL-6, IL-12 ↑ Foxp3 ↑ IL-10	(151)
VitD	MOG MOG^35-55^	TolDC Vaccine: BMDC treated with Adj, loaded with Ag or Ag-mRNA	Therapeutic: i.v.	EAE	↓ Disease Score ↓ Ag-T Response	(156)
VitD	MOG^40-55^	TolDC Vaccine: BMDC treated with Adj, loaded with Ag	Therapeutic: i.v. Prophylactic: i.v.	EAE	↓Disease Score ↓ Ag-response ↑ IL-10 ↑ Treg ↑ Bregs	(153)
VitD	MOG^35-55^	Separate Delivery Vaccine: Adj and Ag	Prophylactic: i.p.	EAE	↓Disease Score ↓ IL-6, IL-17, TNF, IFN-γ ↑ TGF-β ↑ TolDC	(159)
VitD	MOG^35-55^	TolDC Vaccine: BMDC treated with Adj, loaded with Ag	Therapeutic: i.v.	EAE	↓ Disease Score ↓ Th1, Th17 ↑ Bregs ↑ IL-10^+^ T Cells ↑ Tregs ↑ TolDC	(155)
VitD analog	IGRP^206-214^	NP Vaccine: Adj and Ag	Prophylactic: s.c.	T1D	↓ Disease Incidence ↓ TNF, IFN-γ ↑ TolDC	(158)
VitD analog	OVA	Separate Delivery Vaccine: Adj and Ag	Prophylactic: Topical Adj, epicutaneous Ag	DTH	↓ Swelling ↓ Ag-T Response ↓ IFN-γ ↑Tregs	(161)
VitD analog	BDC2.5 mimotope	NP Vaccine: Adj + Ag	Prophylactic: s.c.	T1D	↓ Disease Incidence ↓ INF-γ ↑ Ag-Tregs	(157)
Retinoic acid	MOG	NP/MP Vaccine: Adj + Ag	Prophylactic: s.c	EAE	↓ Disease Score ↓ IL-17A, ↑ Ag-Tr1	(167)

ADA, Antidrug antibody; Adj, Adjuvant; Ag, antigen; BMDC, bone marrow-derived dendritic cells; CD, cluster of differentiation; CIA, collagen-induced arthritis; CII, type II collagen; DTH, Delayed type hypersensitivity; DC, dendritic cell; DNA, deoxyribonucleic acid; EAE, experimental autoimmune encephalomyelitis; EAU, experimental autoimmune uveitis; FOXP3, forkhead box P3; FVIII, Factor VIII; GAD65, glutamic acid decarboxylase; HEL, Hen egg lysozyme; i.d., intradermal; IFN-γ, interferon-γ; IGRP, Islet-specific glucose-6-phosphatase catalytic subunit-related protein; i.m., intramuscular; IRBP, Interphotoreceptor retinoid-binding protein; IL, interleukin; Ins, Insulin; i.p., intraperitoneal; i.v., intravenous(ly); MBP, myelin basic protein; MOG, myelin oligodendrocyte glycoprotein; MP, microparticle; NP, nanoparticle; OVA, ovalbumin; PLP, proteolipid protein; s.c., subcutaneous(ly); T1D, type 1 diabetes; Th; T helper; TGF-β, Transforming Growth Factor-β; TNF, tumor necrosis factor; Tr1, type 1 regulatory T cell; Treg, regulatory T cell; TolDC, tolerogenic dendritic cell.

↓, decrease; ↑, increase.

**Table 3:** Modulators of contact-dependent immune cell signaling.

Adjuvant	Antigen	Formulation	Administration	Disease Model	Major Results	Ref
α-CD3	InsB^9-23^	DNA Vaccine: Adj + Ag vector	Prophylactic: i.v.	T1D	↓ Disease Incidence	(170)
α-CD4 (non-depleting)	FVIII	Separate Delivery Vaccine: Injection of Ag in Alum + Adj	Prophylactic: Anti-CD4 i.p. or i.v. and FVIII s.c. or i.p	ADA	↓ Ag-Antibody	(172)
α-CD4 (depleting)	IRBP^1-20^ arrestin MOG^35-55^	Separate Delivery Vaccine	Therapeutic: i.p.	EAU	↓ Disease Score ↓ Ag-Response ↓ Th1, Th17 ↓ IL-17, IFN-γ ↑ TGF-β, IL-10 ↑ Tregs	(173)
Tregitope	Preproinsulin	NP Vaccine: Adj + Ag	Prophylactic: i.p.	T1D	↓ Disease Incidence	(174)
Tregitope	Preproinsulin	NP Vaccine: Adj-albumin fusion protein + Ag	Therapeutic: s.c.	T1D	↓ Severe Disease Incidence ↑ Mild Disease Reversal	(175)
α-GalCer	InsB^9-23^	NP Vaccine: Adj + Ag	Prophylactic: i.p.	T1D	↓ Disease Incidence ↑ Foxp3	(177)
α-CD40L	FVIII	Co-Delivery Vaccine: Adj + Ag	Prophylactic: i.v.	ADA	↓ Ag-Antibody ↓ Ag-T Cell Responses ↓ IL-2, IL-4, IIFN-γ	(182)
mutant B7.1/CD40L	Proinsulin	DNA Vaccine: encoding membrane bound Ag and Adj fusion protein	Prophylactic: i.m.	T1D	↓ Disease Incidence	(184)
CD40, CD80 and CD86 knockdown	BDC2.5 mimotope	NP Vaccine: encapsulating pCAS9 DNA and CD80, CD86 and CD40 gRNA	Prophylactic: i.v.	T1D	↓ Disease Incidence ↓ IL-17,IFN-γ, IL-6 ↑ IL-10 ↑ Ag-Tregs ↑ TolDC	(183)
LFA-1 peptide (ICAM-1 Inhibitor)	PLP^139-151^	Co-Delivery Vaccine: Adj + Ag on hyaluronic acid backbone	Prophylactic: s.c.	EAE	↓ Disease Score	(186–188)
LFA-1 peptide (ICAM-1 Inhibitor)	PLP^139-151^ MOG^38-50^ GAD65	Conjugate Vaccine: Adj -Ag	Prophylactic: s.c. or i.v.	EAE T1D	↓ Disease Score ↓ Ag-Response ↓ IFN-γ, IL-6 ↓ Th17 ↑ TolDC ↑ Tregs	(189, 190)
α-OX40 (Agonist)	InsB^9-23^	Co-Delivery Vaccine: Adj and Ag	Prophylactic: i.n.	T1D	↓ Disease Incidence ↑ Tregs ↑ IL-10	(192)
PD-L1	MOG^35-55^	TolDC Vaccine: BMDC treated with Adj plasmid and Ag Plasmid	Prophylactic: i.v.	EAE	↓ Disease Score ↓ Ag-Response ↓ Cell Infiltration	(148, 149)
BTLA	MOG^35-55^	TolDC Vaccine: BMDC treated with Adj plasmid treated with Ag load NP	Prophylactic: i.p.	EAE	↓ Disease Score ↓ IFN-γ, IL-2 ↑ Tregs ↑ IL-10, TGF-β	(196)
CD22	FVIII	NP Vaccine: Incorporating Adj +Ag	Prophylactic: i.p.	Hemophilia	↓Bleeding ↓Ag-antibody	(201)
Siglec-GL	OVA HEL	NP Vaccine: incorporating Adj + Ag	Prophylactic: i.v.	Autoantibody	↓ Ag-antibody ↑ Ag-B Cell Responses	(202)

ADA, Antidrug antibody; Adj, Adjuvant; Ag, antigen; BMDC, bone marrow-derived dendritic cells; BTLA, B- and T-lymphocyte attenuator; CD, cluster of differentiation; DTH, Delayed type hypersensitivity; DC, dendritic cell; DNA, deoxyribonucleic acid; EAE, experimental autoimmune encephalomyelitis; EAU, experimental autoimmune uveitis; FOXP3, forkhead box P3; FVIII, Factor VIII; GAD65, glutamic acid decarboxylase; HEL, Hen egg lysozyme; IFN-γ, interferon-γ; IGRP, Islet-specific glucose-6-phosphatase catalytic subunit-related protein; i.m., intramuscular; IRBP, Interphotoreceptor retinoid-binding protein; IL, interleukin; Ins, Insulin; i.p., intraperitoneal; i.v., intravenous(ly); LFA-1, Lymphocyte function-associated antigen 1; MOG, myelin oligodendrocyte glycoprotein; MP, microparticle; NP, nanoparticle; OVA, ovalbumin; PLP, proteolipid protein; s.c., subcutaneous(ly); T1D, type 1 diabetes; Th; T helper; TGF-β, Transforming Growth Factor-β; TNF, tumor necrosis factor; Treg, regulatory T cell; TolDC, tolerogenic dendritic cell.

↓, decrease; ↑, increase.

**Table 4:** Other adjuvants.

Adjuvant	Antigen	Formulation	Administration	Disease Model	Major Results	Ref
O-phospho-L-serine (OPLS)	FVIII	Co-Delivery Vaccine: Adj + Ag	Prophylactic: s.c.	ADA	↓ Ag-antibody ↑ TolDC	(207)
Phosphatidylserine	InsA^21^ InsB^30^	NP Vaccine: Phosphatidylserine-Liposomes with Ag	Prophylactic: i.p.	T1D	↓ Disease Incidence ↓ T Cell Responses ↑ Ag-CD4 T cells	(209)
Phosphatidylserine	FVIII	NP Vaccine: Phosphatidylserine-Liposomes with Ag	Prophylactic: s.c.	ADA	↓ Ag-Antibody	(208)
BAX	GAD55	Co-Delivery DNA Vaccine: DNA-Ag + DNA-Adj	Prophlactic: i.m. Therapeutic: i.d.	T1D	↓Disease Incidence ↓IFN-γ, TNF ↑Tregs	(211) (212)
LPS	MOG^35-55^	TolDC Vaccine: BMDC treated with Adj and loaded with Ag	Therapeutic: i.v.	EAE	↓ Disease Score ↑ CD127^+^ Tregs	(215)
Flagellin B	OVA Der p 2	Co-Delivery Vaccine: Adj + Ag	Prophylactic: i.n.	Allergy	↓ Disease Score ↓ Ag-Antibody ↓ Eosinophils ↓ IL-5, IL-4, IL-13, IFN-γ ↑ IL-10, TGF-β ↑ Tregs ↑ TolDC	(218, 219)
Flagellin B	Der p 2	Fusion Protein Vaccine: Adj-Ag fusion protein	Prophylactic: i.n.	Allergy	↓ Disease Score ↓ Ag-IgE Antibody	(220)
Flagellin A	OVA	Conjugate Vaccine: Adj-Ag	Prophylactic: i.p.	Allergy	↓ Disease Score ↓ Ag-Antibody ↓ IL-6, IL-4, IL-5, IFN-γ ↑ IL-10	(216, 217)
β-glucan	β-cell-Ag	Co-Delivery Vaccine: Adj + Ag	Prophylactic: i.v.	T1D	↓ Disease Incidence ↑ TolDC, Tregs	(224)
Mannan	Grass pollen Ag	Conjugate Vaccine: Adj-Ag	sublingual	Allergy	↑ IgG/IgE ratio ↑ IFN-γ/IL-4 ratio ↑ IL-10 ↑ Tregs	(226)
Mannan	Grass pollen Ag	Conjugate Vaccine: Adj-Ag	Skin prick test (human) Sublingual (mouse)	Allergy	↓ Skin Prick Test Area ↑ IL-10 ↑ Tregs	(225)
Galectin-1	MOG^35-55^	TolDC vaccine: BMDC treated with Adj and loaded with Ag	Therapeutic: i.p.	EAE	↓ Disease Score ↓ IL-17, IFN-γ ↑ IL-10, IL-27	(230)

ADA, Antidrug antibody; Adj, Adjuvant; Ag, antigen; BMDC, bone marrow-derived dendritic cells; CD, cluster of differentiation; DC, dendritic cell; Der p 2, Dermatophagoides pteronyssinus**;** EAE, experimental autoimmune encephalomyelitis; FOXP3, forkhead box P3; FVIII, Factor VIII; GAD65, glutamic acid decarboxylase; IFN-γ, interferon-γ; i.n., intranasal; i.m., intramuscular; IL, interleukin; Ins, Insulin; i.p., intraperitoneal; i.v., intravenous(ly); MOG, myelin oligodendrocyte glycoprotein; NP, nanoparticle; OVA, ovalbumin; PLP, proteolipid protein; s.c., subcutaneous(ly); T1D, type 1 diabetes; Th; T helper; TGF-β, Transforming Growth Factor-β; TNF, tumor necrosis factor; Treg, regulatory T cell; TolDC, tolerogenic dendritic cell.

↓, decrease; ↑, increase.

**Table 5:** Multiple adjuvants.

Adjuvants	Antigen	Formulation	Administration	Disease Model	Major Results	Ref
Dexamethasone + VitD	GAD65	TolDC Vaccine: Monocyte-derived DC treated with Adj and loaded Ag	Prophylactic: i.p.	T1D	↓ Disease Incidence ↓ IL-17, IFN-γ, IL-9, TNF ↑ Il-10 ↑ Tregs	(29)
Dexamethasone + Minocycline	MOG35-55	TolDC Vaccine: BMDC treated with Adj and loaded with Ag	Prophylactic: i.v.	EAE	↓ Disease Score ↓ Ag-T Response ↑ IL-10	(31)
Dexamethasone + abatacept	MOG^35-55^	NP Vaccine: Particles carrying adj + Ag	Therapeutic: s.c. Prophylactic: s.c.	EAE	↓ Disease Score ↓ Cell Infiltration ↓ Th1, Th17 ↑ Tregs	(180)
Dexamethasone + anti-MHC II	MOG^35-55^	Co-Delivery Vaccine: Adj + anti-MHC II nanobody-Ag fusion protein	Therapeutic: i.v.	EAE	↓ Disease Score	(178)
Rapamycin + CD22L	OVA	Co-Administration NP vaccine: LNP with CD22L and AG and separate PGLA NP with Rapamycin or combined	Prophylactic: i.v.	AIA	↓ Disease Incidence ↓ Ag-Antibody ↓ Ag-Plasma Cells ↑ Tregs	(205) (205) (204)
Rapamycin + IL-2/α-IL2 IC	BDC2.5mi	Co-Delivery Vaccine: Adj + Ag	Prophylactic: i.v.	T1D	↓ Disease Incidence ↑ Tregs ↑ IL-10, IL-4	(115)
Rapamycin + IL-2 mutein	HIP6.9 PDC-E2	NP Vaccine: containing Adj + Ag	Prophylactic: i.v.	T1D PBC	↓ Disease Incidence ↓ Disease Score ↓ Pathogenic T Cells ↓ IFN-γ, IL-6 ↑ Tregs ↑ IL-10, IL-4	(114)
Rapamycin + IL-2 fusion protein	MOG	NP Vaccine: containing rapamycin IL-2-α–IL2 fusion proteins and MHC class II/Ag	Therapeutic: i.v. or intra lymph node Prophylactic: i.v. or intra lymph node	EAE	↓Disease Score ↑ Tregs	(116)
IL-2 + Retinoic acid	MOG IRBP	Co-Delivery Vaccine: Adj + Ag	Prophylactic: s.c.	EAE EAU	↓ Disease Incidence ↑ Tr1 Cells ↓ Th17 ↓ IL-17, IFN-γ ↑ CTLA-4^+^ T Cells, IL-10^+^ T Cells	(117)
IFN-β + VitD	MOG^35-55^	TolDC Vaccine: BMDCs treated with VitD and Ag and treatment of mice with IFN-β	Therapeutic: s.c. IFN-β and i.v. TolDCs	EAE	↓ Disease Score ↓ Th17 ↑ IL-10 ↑ Th2	(122)
IL-4 + Retinoic acid	MOG^35-55^	Separate Delivery Vaccine: Free Ad and Adj	Prophylactic: Adj s.c., Ag i.p.	EAE	↓ Disease Score ↓ IL-17, IFN-γ	(168)
IL-10 + α-CD3	Proinsulin GAD65	Probiotic vaccine: L. lactis genetically modified to secrete IL-10 and proinsulin coadministered with α-CD3	Therapeutic: α-CD3 i.v. and intragastric inoculation of probiotic	T1D	↓ Reversed Disease ↓ IFN-γ ↑ IL-10 ↑ CTLA-4^+^ Tregs ↑ Tregs	(104–106)
IL-10 + TGF-β	CII	TolDC Vaccine: BMDC treated with Adj and loaded with Ag	Therapeutic: i.v.	CIA	↓ Disease Score ↓ Autoantibody ↓ IL-17, IFN-γ, IL-6, TNF ↑ TGF-β, IL-10 ↑ Tregs	(86)
IL-10 + TGF-β	FVIII	TolDC Vaccine: BMDC treated with Adj and loaded with Ag	Prophylactic: i.v.	ADA	↓ Ag-antibody	(87)
TGF-β + Retinoic acid	InsB^9-23^	NP Vaccine: Containing Adj + Ag	Prophylactic: s.c.	T1D	↓ Disease Incidence	(93)
GM-CSF + CpG	Ins	MP Vaccine: Co-Delivered MP loaded with Ag and hydrogel loaded with Adj	Prophylactic: s.c.	T1D	↓ Disease Incidence ↓ Protein Urea ↑ IL-10	(131)
FasL + MCP-1	MOG^35–55^ GAD^524–543^	MP Vaccine: loaded with MCP-1, surface FasL and Ag	Therapeutic: i.v.	T1D EAE	↓ Disease Incidence ↓ Disease Score ↓ Pathogenic T Cells ↓ IFN-γ, IL-17, TNF, IL-6 ↑ Ag-Tregs	(197)
Leflunomide + phosphatidylserine	CII^250-270^	NP Vaccine: Co-Delivery of Leflunomide Ag in Phosphatidylserine Lipid NPs.	Therapeutic: i.v.	CIA	↓Disease Score ↑Tregs	(210)
Dexamethasone + GM-CSF + VitD	Human proteoglycan	TolDC Vaccine: BMDCs treated with adj and Loaded with Ag	prophalactic: i.v.	AIA	Disease reducetion was antigen independent.	(27)
Dexamethasone + cobalt (III) protoporphyrin (CoPP) + Rosiglitazone	Histone	TolDC Vaccine: BMDC were treated with adjuvants and loaded with ag	Prophylactic: i.v.	SLE	↓ Disease Score ↓ Protein Urea ↓ Ag-Antibody	(33)
Dexamethasone + MPLA + VitD	GAD65	TolDC Vaccine: BMDC treated with Adj + Ag	Prophylactic: i.p.	T1D	↓ Disease Incidence *antigen-loading reduced TolDC suppressive effects	(30)
GM-CSF + TGF-β + VitD	MOG^35-55^	MP Co-Delivery Vaccine: 1μM MP loaded with Ag or VitD and 30 μM particles loaded with GM-CSF or TGF-β.	Therapeutic: s.c. Prophylactic: s.c.	EAE	↓ Disease Score ↓ IL-17, GM-CSF, IL-6, IL-12, TNF ↑ TolDC	(89, 90)
GM-CSF + TGF-β + VitD	Ins	MP Co-Delivery Vaccine: 1μM MP loaded with Ag or VitD and 30 μM particles loaded with GM-CSF or TGF-β.	Therapeutic: s.c. Prophylactic: s.c.	T1D	↓ Disease Incidence ↑ Tregs ↑ TolDC	(88, 91, 92)
IL-10 + IL-2 + TGF-β	Preproinsulin	DNA Vaccine: Single plasmid encoding Ag and Adj	Prophylactic: i.m.	T1D	↓ Disease Incidence ↓ IL-6 ↑ IL-10	(95)
Dexamethasone + CpG + SC-514 + Simvastatin	MOG^35-55^	NP Vaccine: containing Adj + Ag	Prophylactic: i.p.	EAE	↓ Disease Score ↓ Ag-T Cells ↓ Ag-Antibody ↑ Ag-Tregs ↑ TolDC	(32)
TGF-β + α-Fas + CD47Fc+ PD-L1Fc	MOG^40–54^ MOG^35–55^	NP Vaccine: MOG40–54/H-2Db-Ig dimer, MOG35–55/I-Ab multimer, anti-Fas, PD-L1-Fc and CD47-Fc and encapsulating transforming growth factor-β1	Therapeutic: i.v.	EAE	↓ Disease Score ↓ T Cell Infiltration ↓ Th1, Th17 ↑ Tregs ↑ IL-10, TGF-β ↑ Apoptotic T Cells	(198, 199)

ADA, Antidrug antibody; AIA, antigen-induced arthritis; Adj, Adjuvant; Ag, antigen; BMDC, bone marrow-derived dendritic cells; CD, cluster of differentiation; CIA, collagen-induced arthritis; CII, type II collagen; DC, dendritic cell; DNA, deoxyribonucleic acid; EAE, experimental autoimmune encephalomyelitis; EAU, experimental autoimmune uveitis; FOXP3, forkhead box P3; FVIII, Factor VIII; GAD65, glutamic acid decarboxylase; HEL, Hen egg lysozyme; HIP, Hsp70-interacting protein**;** IFN-γ, interferon-γ; i.m., intramuscular; IL, interleukin; Ins, Insulin; i.p., intraperitoneal; i.v., intravenous(ly); IRBP, Interphotoreceptor retinoid-binding protein; MOG, myelin oligodendrocyte glycoprotein; MP, microparticle; NP, nanoparticle; OVA, ovalbumin; s.c., subcutaneous(ly); PDC-E2, E2 component of the pyruvate dehydrogenase complex; SLE, systemic lupus erythematosus; T1D, type 1 diabetes; Th; T helper; TGF-β, Transforming Growth Factor-β; TNF, tumor necrosis factor; Tr1, type 1 regulatory T cell; Treg, regulatory T cell; TolDC, tolerogenic dendritic cell.

↓, decrease; ↑, increase.

**Table 6:** Human clinical trials.

Adjuvant	Antigen	Formulation	Rout	Disease	Phase	Major Results	PMID/Trial Number
Dexamethasone	MBP^13–32^ MBP^83–99^ MBP^11–129^ MBP^146–170^ MOG^1–20^ MOG^35–55^ PLP^139–154^	TolDC Vaccine: Monocyte-derived DC treated with Adj and loaded with Ag	i.v.	MS	Phase I	↑Tr1	(34) NCT02283671
Dexamethasone + VitD	Proinsulin (C19-A3)	TolDC Vaccine: Monocyte-derived DCs treated with Adj and loaded with Ag	i.d.	T1D	Phase I	↓ Ag-CD8+ T Cells ↓ INF-γ ↑ ICOS^+^CCR4^+^TIGIT^+^ Tregs No change in Autoantibody ↑ Uricase Activity	(164) 2013-005476-18
Dexamethasone + VitD + MPLA	autologous Synovial Fluid	TolDC Vaccine: Monocyte-derived DC treated with Adj and loaded with Ag	intra-articular	RA	Phase I	- No clinical effects were detectable.	(35) NCT01352858
Rapamycin	Pegadricase	NP/Co-Delivery Vaccine: NP with rapamycin alongside IV pegadricase	i.v.	Gout	Phase Ia, Ib and III	↓ Ag-antibody ↓ Uric Acid Levels ↑ Uricase Activity	(53, 54) NCT02464605 NCT04513366 NCT02648269 NCT04596540
Rapamycin	AAV8	NP/Co-Delivery Vaccine: Rapamycin NP given alongside IV AAV8 vector	i.v.	ADA	Phase I	↓Ag-antibody	press release
Bay11-7082	Citrullinated aggrecan, vimentin, CII, and fibrinogen peptides	TolDC Vaccine: Monocyte-derived DC treated with Adj and loaded with Ag	i.d.	RA	Phase I	↓ T Effector ↓ IL-15 ↓ IL-6 ↓ IL-29 ↓ CX3CL1 ↓CXCL11 ↑ Tregs	(64) CTRN12610000373077
VitD	GAD65	Separate Vaccine: Ag in alum and oral VitD	Ag-alum intra-lyphatic, oral vitamin D	T1D	Phase I-III	- Stable Beta Cell Function - Stable Metabolic Control	(162) NCT04262479NCT050 18585NCT03345004NCT02352974
VitD	MBP^13-32^ MBP^111-129^ MBP^154-170^ PLP^139-154^ MOG^1-20^ MOG^35-55^ MBP^83-99^	TolDC Vaccine: Monocyte-derived DC treated with Adj and loaded with Ag	i.d.	MS	Phase I	N/A	(163) NCT02618902 and NCT02903537
VitD analog	CII^259-273^	NP Vaccine: LNP encapsulating Adj + Ag	s.c	RA	Phase I	↓ Ag T Cells ↓ Memory B Cells ↑ PD1^+^ T Cells	(165) ACTRN12617001482358
TGF-β1 + IL-10 + IL-2	Preproinsulin	DNA Vaccine: Adj + Ag plasmid,	s.c.	T1D	Phase I	N/A	NCT04279613
IL-10 + α-CD3	Preproinsulin	Probiotic Vaccine: L. lactis bacteria carrying encoding Ag + Adj	oral probiotic and i.v. infusions of teplizumab (α-CD3)	T1D	Phase Ib and IIa	↑ Metabolic Improvement ↑ Exhausted CD8^+^ T Cells ↓ Ag-CD8^+^ T Cells	NCT03751007 (107)
Mannan	Grass pollen Ag	Conjugate Vaccine: Adj-Ag	s.c. & sublingual	Allergy	Phase II	↓ Nasal Provocation	(227) NCT02654223
Mannan	Dust mite pollen Ag	Conjugate Vaccine: Adj-Ag	s.c. & sublingual	Allergy	Phase II	↓ Nasal Provocation	(228) NCT02661854

ADA, Antidrug antibody; Adj, Adjuvant; Ag, antigen; CD, cluster of differentiation; DC, dendritic cell; DNA, deoxyribonucleic acid; GAD65, glutamic acid decarboxylase; i.d., intradermal; IFN-γ, interferon-γ; i.m., intramuscular; IRBP, Interphotoreceptor retinoid-binding protein; IL, interleukin; Ins, Insulin; i.v., intravenous(ly); MOG, myelin oligodendrocyte glycoprotein; NP, nanoparticle; PLP, proteolipid protein; s.c., subcutaneous(ly); RA, rheumatoid arthritis; T1D, type 1 diabetes; Th, T helper; TGF-β, Transforming Growth Factor-β; TNF, tumor necrosis factor; Treg, regulatory T cell; TolDC, tolerogenic dendritic cell.

↓, decrease; ↑, increase.

General immunosuppressive agents include molecules with broad immunosuppressive effects on multiple aspects of the immune system, often by suppressing intracellular signaling pathways to reduce inflammation-induced gene expression (**Figure 1B**). General immunosuppressive tolerogenic adjuvants are used both to promote tolerogenic features on antigen presenting cells (APCs) and to suppress effector T and B cell function.

Cytokines orchestrate the balance between immune activation and regulation, making them valuable tools as tolerogenic adjuvants to shape antigen-specific immune responses. Classical anti-inflammatory cytokines like transforming growth factor β (TGF-β), and interleukin (IL)-10 exert potent immunosuppressive functions affecting many aspects of the immune system, including the capacity to induce tolDCs and Tregs (**Figures 1C, D**). In addition to classical anti-inflammatory cytokines, cytokines affecting specific immune cell subsets or skewing immune balance can also be harnessed as tolerogenic adjuvants.

Many vitamins are important for maintaining a healthy immune system. Vitamins A and D signal in a hormone-like manner via nuclear receptors and are potent immunoregulators (10). Some of their immunomodulatory effects are inhibition of T effector cell activation and promotion of tolerogenic APCs (**Figures 1C, D**).

Modulating the immune synapse, the dynamic interface between APCs and T cells during antigen recognition, offers a promising approach to inducing immune tolerance (**Figure 1D**). By manipulating the immune synapse, one can modify T cell receptor (TCR) signaling strength to skew T cell responses or induce anergy or apoptosis (11). In addition to TCR signaling, modulation of APC co-stimulatory or co-inhibitory molecules in the immunological synapse can also be utilized to modify the outcome of APC-T cell interactions (12). (**Figures 1D, E**).

In addition to the adjuvant types described above, there are several other types of adjuvants. Described here are apoptotic remnant mimics (**Figure 1F**), Toll-like receptor (TLR) agonists and glycans and glycan-binding proteins which may promote tolerogenic immune responses.

Depot adjuvants and delivery systems including cell targeting moieties and nanoparticles/microparticles inherently act as adjuvants since they enhance the immune responses to incorporated antigens (2, 3, 13, 14). Cell targeting strategies which promote antigen delivery to dendritic cells often have tolerogenic properties (3, 14) and can be considered tolerogenic adjuvants. However, in this review we specifically explore adjuvants with tolerogenic properties that actively modify the immune milieu to facilitate shifts in immune cell subsets and/or phenotypes.

## 2 Tolerogenic adjuvants

### 2.1 Immunosuppressive agents

#### 2.1.1 Dexamethasone

Glucocorticoids are potent anti-inflammatory and immunosuppressive agents used for treatment of autoimmune and inflammatory conditions (15–17). They mediate their effects through engagement with the nuclear glucocorticoid receptor inducing transcriptional regulation or rapid non-genomic effects (15, 17). In immune cells, the synthetic glucocorticoid dexamethasone suppresses production of most cytokines while increasing production of IL-10, inhibits lymphocyte activation and promotes lymphocyte apoptosis (15). Glucocorticoids dampen overall T cell activation by interfering with TCR signaling, and evidence suggests that glucocorticoids preferentially suppresses Th1 and Th17 T cells. Glucocorticoid treatment is also associated with increased circulating Tregs (15). In APCs, glucocorticoids induce tolerogenic features including attenuated DC maturation and reduced expression of MHC class II and co-stimulatory molecules (15, 18).

Tolerogenic vaccines consisting of antigen delivered with dexamethasone have been successful in murine models of experimental autoimmune encephalitis (EAE) (19–22), atherosclerosis (23), antigen-induced arthritis (AIA) (24) and type 1 diabetes (T1D) (25). Other tolerogenic vaccines use dexamethasone to induce tolDCs, which when loaded with disease relevant antigens and injected reduced disease in models of arthritis (26, 27), dust mite allergy (28) and T1D (29). The disease inhibition was often accompanied by an increase in Tregs over effector T cells and increase in anti-inflammatory cytokines. However, tolDC-mediated disease suppression in one arthritis model was independent of antigen (27) and antigen-loaded dexamethasone-derived tolDCs exacerbated a model of T1D while unloaded tolDCs suppressed disease (30) (**Table 1**).

Dexamethasone is often used together with other adjuvants to induce tolDCs. TolDC vaccines of dexamethasone in combination with one or more other adjuvants suppressed disease in models of EAE (31, 32) and systemic lupus erythematosus (SLE) (33) (**Table 5**).

Dexamethasone-containing tolerogenic vaccines have been investigated for human use. A phase I clinical trial investigated transfer of antigen-loaded dexamethasone-treated monocyte-derived DCs for treatment of multiple sclerosis and neuromyelitis optica spectrum disorders, by intravenous injection of tolDC every two weeks for a total of three doses. The tolDC vaccine was well tolerated and there was an increase of regulatory Tr1 cells at 12 weeks of follow-up (34). In another phase I trial, antigen-loaded autologous monocyte-derived DCs treated with a combination of dexamethasone, the vitamin D derivative calcitriol, and the TLR4 agonist MPLA were administered into inflamed knee joints. The treatment was well tolerated but did not result in reduction in disease severity or consistent immunoregulatory features (35) (**Table 6**).

#### 2.1.2 Rapamycin

Rapamycin is a small molecule inhibitor of mTOR, a kinase that regulates cell growth and metabolism. Immunosuppressive effects of rapamycin includes suppression of the activation and proliferation of conventional T cells, promotion of T cell anergy or deletion, and enhancement of the development and function of Tregs (36). Additionally, rapamycin can inhibit the differentiation and maturation of DCs and promote tolDC features (37). Rapamycin is clinically used as an immunosuppressant for prevention of organ transplant rejection, as well as an anti-cancer drug.

Tolerogenic nanoparticle vaccines with antigen and rapamycin have successfully reduced disease in multiple models of autoimmunity including arthritis (38), Alzheimer’s disease (39), vitiligo (40), allergic airway disease (41, 42), T1D (43), and EAE (44, 45). Rapamycin-containing nanoparticle vaccines also prevented anti-drug antibody responses toward coagulation factor VIII (FVIII) (45, 46), uricase (47), adalimumab (anti-TNFα) (47), and adenoviral vectors used in gene therapy (48–50). Administration of rapamycin without nanoparticle carrier suppressed OVA-induced skin graft rejection (51) and anti-FVIII anti-drug antibodies in mice (52). Observed immunological alterations in studies of tolerogenic vaccines with rapamycin included increased Tregs, decreased levels of inflammatory cytokines, and/or decreased co-stimulatory molecules on DCs (**Table 1**).

Two phase Ia and phase Ib clinical trials were conducted investigating administration of rapamycin together with pegadricase, a biological drug used for the treatment of gout, finding that rapamycin suppressed the development of anti-drug antibodies in a dose dependent manner (53). Their following phase III trial demonstrated high response rate, safety, and clinically meaningful reduction in serum urate (54). Another phase I clinical trial administered rapamycin-containing nanoparticles together with AAV8 capsid used in adenoviral gene therapies, leading to reduced development of anti-AAV8 antibodies compared to subjects receiving capsid without rapamycin (press release) (**Table 6**).

#### 2.1.3 Calcineurin inhibitors

Cyclosporine A and FK506 (tacrolimus) are calcineurin inhibitors which suppress downstream nuclear factor of activated T cell (NFAT) signaling and IL-2 production (55). Tolerogenic vaccination with cyclosporine A and autoantigens prevented the development of T1D in pre-diabetic mice and promoted Tregs and tolDCs (56). Likewise, tolerogenic vaccination with FK506 co-delivered with DNA encoding autoantigen suppressed EAE (57), and adoptive transfer of antigen-loaded FK506-induced tolDCs reduced disease in a model of CIA (58), in both studies with a reduction in Th17 responses (**Table 1**).

#### 2.1.4 NFκB inhibitors

Nuclear factor kappa B (NFκB) is a transcription factor inducing expression of pro-inflammatory genes including cytokines, cell adhesion molecules, and immunoreceptors. Inhibition of NFκB suppresses T cell responses and induces immunoregulatory features in APCs (59).

Tolerogenic vaccination with antigen-loaded tolDCs treated with NFκB inhibitor BAY 11-7082 suppressed disease in AIA (60), and antigen-loaded tolDCs treated with NFκB inhibitor andrographolide reduced anti-drug antibodies in hemophilia A mice (61). A nanoparticle vaccine loaded with antigen and A20, an anti-inflammatory protein inhibiting NF-κB activation (62), suppressed Th2 responses and reduced disease in an asthma model (63) (**Table 1**).

A phase I clinical trial investigated antigen-loaded autologous Bay11-7082-treated tolDCs in rheumatoid arthritis. The vaccine reduced effector T cells and increased Tregs, together with a reduction in inflammatory cytokines. The treatment did not induce disease flares and lead to decreased rheumatoid arthritis DAS28 score (64) (**Table 6**).

#### 2.1.5 Kynurenine and AhR agonists

Kynurenine is an immunoregulatory tryptophan metabolite signaling via the aryl hydrocarbon receptor (AhR). AhR signaling can influence T cell differentiation and function of APCs to favor expansion of Tregs (65, 66). Tolerogenic vaccination with nanoparticles loaded with autoantigen and the AhR agonist ITE (2-(1′H-indole-3′-carbonyl)-thiazole-4-carboxylic acid methyl ester), suppressed disease in models of EAE (67, 68) and T1D (69), and tolerogenic vaccination with kynurenine and antigen-expressing phages prevented hyperglycemia in a model of T1D (70) (**Table 1**).

#### 2.1.6 Other immunosuppressive agents

Janus kinase (JAK) and signal transducer and activator of transcription proteins (STAT) signaling induces immune cell activation and cytokine production (71). Tolerogenic vaccination with autoantigen-loaded tolDCs treated with JAK/STAT inhibitors tofacitinib and BD750 suppressed disease, reduced Th1 and Th17 responses, and increased Tregs in models of EAE (72, 73) (**Table 1**).

Rosiglitazone is an anti-diabetic drug that activates peroxisome proliferator–activated receptor gamma (PPARγ). In immune cells, rosiglitazone can inhibit inflammatory cytokines and promote tolerogenic APCs (74). TolDC vaccination with antigen-loaded, rosiglitazone-treated tolDCs suppressed disease in CIA (75) (**Table 1**), and autoantigen-loaded tolDCs treated with rosiglitazone in combination with dexamethasone and cobalt (III) protoporphyrin suppressed a murine model of SLE (33) (**Table 5**).

Inhibition of the protein kinase glycogen synthase kinase 3 (GSK-3) in immune cells leads to reduced inflammatory cytokine production and increased IL-10 (76). Vaccination with antigen-loaded tolDCs treated with GSK-3β inhibitor K313 suppressed disease in EAE (77) (**Table 1**).

Prostaglandin I2 (PGI2) is a lipid signaling mediator most known for its vasodilating and anti-thrombotic effects. PGI2 also has anti-inflammatory properties and protective effects in allergy and asthma (78, 79). Tolerogenic vaccination with antigen-loaded tolDCs treated with PGI2 analog iloprost reduced disease in ovalbumin-induced asthma (80). Additionally, direct delivery of iloprost and antigen using a hydrogel suppressed antigen-induced lung inflammation and increased the frequency of antigen-specific Tregs (80) (**Table 1**).

### 2.2 Cytokines and chemokines

#### 2.2.1 TGF-β

TGF-β is a strongly immunosuppressive cytokine, because genetic deficiency of TGF-β leads to fatal autoimmunity (81). TGF-β is an immunosuppressive cytokine with multiple effects on the immune system: it promotes Treg development and function, inhibits B and T cell proliferation, suppresses differentiation of Th1 and Th2 cells, and induces tolDCs (82). However, when combined with specific other cytokines, TGF-β may trigger T cells to differentiate into non-regulatory phenotypes such as Th17 effectors in presence of IL-6 and Th9 in presence of IL-4 (82).

A tolerogenic nanoparticle vaccine containing autoantigen and TGF-β reduced disease and immune cell activation in EAE (83). TolDC vaccination with autoantigen-loaded tolDCs cultured in presence of TGF-β or TGF-β receptor agonist suppressed disease in a CIA model (84) and reduced anti-drug antibodies toward FVIII (85), together with increases in Tregs and IL-10 (**Table 2**).

In combination with other adjuvants, tolerogenic vaccines using both TGF-β and IL-10 suppressed disease in CIA (86) and reduced anti-drug antibodies toward FVIII (87). Tolerogenic vaccination with microparticles containing GM-CSF and TGF-β1 alongside nanoparticles with antigen and vitamin D suppressed EAE and T1D (88–92) and microparticles loaded with TGF-β, retinoic acid, and autoantigens suppressed T1D (93) (**Table 5**).

A tolerogenic DNA vaccination with autoantigen-encoding plasmids in combination with plasmids for either TGF-β, IL-10, and or IL-2 suppressed disease in EAE (94) and T1D (95) (**Table 5**). A phase I clinical trial is registered for this tolerogenic DNA vaccine to evaluate vaccine safety in patients with T1D (**Table 6**).

#### 2.2.2 IL-10

IL-10 is a powerful immunosuppressive and anti-inflammatory cytokine, absence of which causes spontaneous colitis in mice (96). IL-10 suppresses antigen presentation and inflammatory cytokine production by APCs and simultaneously increases their release of anti-inflammatory mediators. In CD4^+^ T cells, IL-10 inhibits proliferation and cytokine production and promotes the development of regulatory Tr1 cells (97).

Tolerogenic vaccination with antigen-loaded tolDCs engineered to express IL-10 suppressed disease in mouse models of T1D and asthma (98, 99), and antigen-loaded tolDCs cultured in presence of IL-10 reduced disease in EAE (100). Tolerogenic vaccination with nanoparticles containing IL-10 and antigen suppressed disease in EAE (101), and DNA vaccines encoding IL-10 and antigen suppressed disease in EAE and T1D models (102, 103) (**Table 2**).

A probiotic vaccine of *Lactococcus lactis (L. lactis)* genetically engineered to secrete IL-10 and pro-insulin administered together with anti-CD3 ameliorated disease and increase Tregs in models of T1D (104–106) (**Table 5**). The *L. lactis* probiotic vaccine has been studied in human clinical trials where results from phase Ib and IIa studies demonstrated treatment to be safe, metabolic variables were either stabilized or improved, and antigen-specific CD8^+^ T cells were reduced (107) (**Table 6**).

#### 2.2.3 IL-2

IL-2 mediates T cell survival, differentiation, and proliferation. IL-2 is specifically required for Treg homeostasis and suppression of autoimmunity and genetic deletion results in systemic autoimmunity in mice (108). In addition, recent studies showed that low-dose IL-2 treatment induces the expansion of Treg cells and had efficacy in numerous mouse models and some early efficacy in clinical trials of T1D, graft-vs-host disease and SLE. Different types of tolerogenic vaccination with IL-2 treatment in combination with antigen exposure suppressed disease in models of EAE (109), experimental autoimmune uveitis (EAU) (110, 111), T1D (112), delayed-type hypersensitivity (DTH) (112), and reduced development of anti-drug antibodies toward FVIII in hemophilia A (113). Overall, the vaccines led to increased Tregs and anti-inflammatory cytokines (**Table 2**).

Tolerogenic vaccines using IL-2 in combination with rapamycin expanded Tregs and suppressed disease in models of T1D (114, 115), EAE (116), and primary biliary cholangitis (114). Furthermore IL-2 in combination with Retinoic Acid suppressed EAE and EAU (117). These combination vaccines expanded Tregs or induced antigen-specific Tr1 cells (**Table 5**).

#### 2.2.4 IFN-β

Interferon beta (IFN-β) is a type I interferon with immunomodulatory properties, used therapeutically for multiple sclerosis (MS). IFN-β reduces T cell activation, promotes Tregs and induces tolDCs (118, 119). Tolerogenic vaccines comprised of autoantigen and IFN-β suppressed murine and rat models of EAE via the induction of neuroantigen-specific, suppressive CD25^+^ Tregs (120, 121) (**Table 2**). In addition, mice treated with IFN-β while receiving autoantigen-loaded vitamin D-treated tolDCs, further suppressed disease in a model of EAE (122) (**Table 5**).

#### 2.2.5 GM-CSF

In addition to being a growth factor and chemokine, GM-CSF possesses anti-inflammatory effects. Administration of GM-CSF leads to a reduction in disease severity in several animal models of autoimmune disease (123), and GM-CSF promotes development and function of both tolDCs and Tregs (123, 124).

Tolerogenic vaccines with antigen-GM-CSF conjugates using neuropeptide autoantigens have been used to treat EAE in mice and rats, accompanied by increased Tregs (125–129). Additionally, GM-CSF is used for differentiation of DCs for most tolDC vaccines. Although most tolDC vaccines use additional adjuvants, also without other adjuvants transfer of antigen-loaded GM-CSF differentiated tolDCs suppressed murine EAU (130) (**Table 2**). A hydrogel/microparticle vaccine incorporating GM-CSF and TLR9 agonist CpG with autoantigen suppressed disease in a model of T1D (131) (**Table 5**).

#### 2.2.6 Other cytokines and chemokines

IL-35 is a potent inducer of Tregs and regulatory B cells, and it can inhibit the proliferation and function of effector Th1 and Th17 cells. IL-35 has been shown to be protective against autoimmune disease and IL-35 treatment have been able to suppress disease in multiple models of autoimmunity and chronic inflammation (132). In tolerogenic vaccines, tolDCs engineered to overexpress IL-35 and loaded with disease relevant antigen suppressed EAE and DTH (133, 134) (**Table 2**).

IL-27 is an immunoregulatory cytokine which can promote tolerance by supporting development of Tregs and Tr1, antagonizing development of Th2 and Th17 cells, and by increasing co-inhibitory receptor expression on APCs (135). Tolerogenic vaccination by adoptive transfer of IL-27-conditioned antigen-loaded DCs led to a significant amelioration of disease and reduction in Th1 and Th17 cells in murine EAE (136) (**Table 2**).

IL-4 promotes type 2 immunity and suppresses Th1 polarization. Treatment with IL-4 suppressed disease severity in models of EAE and arthritis (137, 138). Tolerogenic DNA vaccines encoding IL-4 and antigen suppressed murine models of CIA, EAE, and T1D (139–142) (**Table 2**).

Hepatocyte growth factor (HGF) is a cytokine with pleiotropic effects, including the promotion of tolDCs (143). In a model of EAE, systemic HGF ameliorated disease and tolerogenic vaccination with HGF-treated antigen-loaded DCs mediated functional recovery in mice with established EAE and suppressed T cell mediated inflammation (144) (**Table 2**).

Vasoactive intestinal peptide (VIP) is a peptide functioning as a neurotransmitter in the central and peripheral nervous systems and has multiple effects, including immune modulation. VIP reduces the release of inflammatory cytokines, stimulates production of IL-10 and TGF-β, and decreases the co-stimulatory activity of APCs (145). Tolerogenic vaccination with antigen-loaded VIP-treated tolDCs led to amelioration of CIA and EAE, accompanied by increased levels of Tr1 cells (146) (**Table 2**).

TNF-related apoptosis-inducing ligand (TRAIL) is a cytokine that induces apoptosis and activation of NFκB. TRAIL has immunoregulatory effects demonstrated by the exacerbated development of autoimmunity in TRAIL-deficient mice (147). Tolerogenic vaccination with DCs engineered to co-express TRAIL and antigen reduced antigen-specific T cell responses and disease symptoms in models of EAE (148, 149) (**Table 2**).

#### 2.2.7 Cytokine silencing

Just as the addition of anti-inflammatory or immunoregulatory cytokines can promote tolerogenic responses, silencing of inflammatory cytokines can also be effective. Silencing of B cell activating factor (BAFF), an essential cytokine for both T and B cell activation (150), in TolDCs using siRNA suppressed murine CIA and promoted Tregs (151) (**Table 2**).

### 2.3 Vitamins and vitamin derivates

#### 2.3.1 Vitamin D

Vitamin D is primarily known for its role in calcium homeostasis and bone health, but it is also immunoregulatory. Having low levels of vitamin D is associated with increased susceptibility to a variety of infectious and autoimmune diseases. Vitamin D suppresses T cell activation, skews T cell differentiation away from Th17 while promoting Tregs and Th2 responses, and promotes tolerogenic DC features, including low surface expression of co-stimulatory molecules and decreased production of inflammatory cytokines (10, 152). Vitamin D signals via a nuclear receptor and exert immunoregulatory effects by regulating gene expression (10).

Many tolerogenic vaccines using vitamin D are tolDC vaccines. Vitamin D-treated tolDCs loaded with antigen or antigen-encoding mRNA reduced disease and promoted immunoregulatory cells and cytokines in murine models of EAE (153–156). The vitamin D-treated tolDCs had reduced expression of MHC class II, co-stimulatory molecules, and pro-inflammatory cytokines, and induced less T cell proliferation compared to DCs that were untreated (153, 154) (**Table 2**). TolDC vaccines using a combination of vitamin D and dexamethasone suppressed murine models of arthritis (26) and T1D (29) (**Table 5**).

Tolerogenic nanoparticle vaccines with vitamin D have been examined in T1D (157, 158), where the nanoparticles reduced disease incidence and increased Tregs or tolDCs *in vivo*. Separate delivery studies of vitamin D and antigen have been studied in EAE (159, 160) and DTH (161), in all studies reducing disease severity and inflammation (**Table 2**).

Vitamin D-containing tolerogenic vaccines have been investigated in human clinical trials. In a phase IIa trial of latent autoimmune diabetes in adult patients were treated with daily oral Vitamin D and monthly injections of antigen-alum. The trial demonstrated safety and stable β-cell function and metabolic control at 5 months follow-up (162). Two phase I clinical trials have been registered to test tolerogenic vaccination with antigen-loaded vitamin D-treated monocyte-derived DCs in multiple sclerosis (163). Likewise, a phase I clinical trial tested monocyte-derived DC loaded with proinsulin and treated with Vitamin D and dexamethasone in T1D. The study showed the treatment was safe and lead to reduce proinsulin specific CD8+ T cells and increased ICOS^+^ CCR4^+^ TIGIT^+^ Tregs (164). Another phase I trial investigated liposomes containing collagen II and calcitriol, the active form of vitamin D, for the treatment of rheumatoid arthritis. The calcitriol-antigen-liposomes led to reduced pathogenic T cells and expansion of antigen-specific PD1^+^ T cells (165) (**Table 6**).

#### 2.3.2 Retinoic acid

Retinoic acid is an immunoregulatory vitamin A metabolite, which like vitamin D signals via a nuclear receptor (10). Retinoic acid promotes the development and function of Tregs while inhibiting the differentiation and activation of effector Th1 and Th17 cells. DCs and macrophages can produce retinoic acid to support Treg induction and maintenance, and the retinoic acid-producing capacity of DCs is further enhanced upon retinoic acid exposure (10, 166).

Tolerogenic vaccination with liposomes incorporating retinoic acid and autoantigen converted pathogenic autoantigen specific Th17 cells to Tr1 cells and suppressed disease in EAE (167). Tolerogenic vaccination with retinoic acid, IL-2, and autoantigen suppressed EAE and EAU and pathogenic Th17 and Th1 responses (117). Prophylactic tolerogenic vaccination with retinoic acid, TGF-β and autoantigen inhibited the incidence of T1D in mice (93), and in another study treatment of mice with autoantigen and retinoic acid in combination with IL-4 suppressed EAE (168) (**Tables 2**, **6**).

### 2.4 Modulators of contact-dependent immune cell signaling

#### 2.4.1 T cell modulation

CD3 is the invariant chain of the TCR. Anti-CD3 monoclonal antibodies suppresses disease in numerous animal models of autoimmunity, and anti-CD3 is an FDA approved treatment to delay early onset type 1 diabetes. The exact mechanism of anti-CD3-mediated immune suppression is unclear but proposed mechanisms include prevention of T cells from recognizing their antigens and the induction of anergy or apoptosis in activated T cells while sparing Tregs (169). Tolerogenic vaccination with anti-CD3 treatment in combination with an antigen-expressing lentiviral vector suppressed T1D and induced autoantigen-specific Tregs (170). Anti-CD3 is also a component of the previously described probiotic vaccine (104–106) (**Tables 3**, **5**).

CD4 is a glycoprotein on helper T cells which primarily functions as a TCR co-receptor. Non-depleting anti-CD4 therapy has been shown to suppress autoimmunity and graft rejection by modulating the function of CD4^+^ T cells by blocking T cell activation and promoting Treg differentiation and suppressor functions (171). A tolerogenic vaccine comprised of aluminum hydroxide (alum), FVIII, and non-depleting anti-CD4 prevented development of anti-drug antibodies in mice (172). Another tolerogenic vaccine using treatment with depleting anti-CD4 antibodies followed by antigen administration suppressed disease in a murine model of EAU via the induction of antigen-specific Tregs (173) (**Table 3**).

“Tregitopes” are peptides derived from IgG that are recognized by a subset of natural Tregs. When presented in MHCII, these peptides activate Tregitope-specific Tregs and suppression of effector T cell responses to co-delivered antigens. Administration of nanoparticles with antigen and tregitopes decreased incidence and severity of T1D in mice (174). Likewise, co-administration of autontigen with Tregitope-albumin fusion proteins decreased incidence and reverse mild T1D (175) (**Table 3**).

Invariant natural killer T cells (iNKT cells) are immunoregulatory T cells important for preventing autoimmune reactions. The glycolipid α-galactosylceramide (α-GalCer) is a strong inducer of iNKT cells and has been shown to suppress disease in multiple animal models of autoimmunity (176). A tolerogenic vaccine comprised of lipid nanoparticles carrying autoantigen and α-GalCer prevented the development of diabetes in prediabetic mice (177) (**Table 3**).

#### 2.4.2 Modulation of the immunological synapse

Including MHC class II-targeting molecules in a tolerogenic vaccine ensures delivery to APCs and may disrupt the immunological synapse. A tolerogenic vaccine with antigen conjugated to antibody fragments (nanobodies) targeting MHC class II suppressed disease in EAE. When combined with dexamethasone, the vaccine also overcame the inflammation associated with antigen exposure (178) (**Table 5**).

Co-stimulatory signals, such as CD80 and CD86, are necessary for T cell activation by APCs. T cell recognition of antigen on MHC II without co-stimulation results in anergy or apoptosis. Abatacept, a cytotoxic T-lymphocyte associated protein 4 (CTLA-4)-Fc fusion protein blocks CD80 and CD86 and is FDA approved for the treatment of autoimmune arthritis (179). Tolerogenic vaccination with nanoparticles displaying abatacept and carrying autoantigen and dexamethasone suppressed EAE (180) (**Table 5**).

Signaling via CD40-CD40L induces activation and pro-inflammatory cytokine production in both B cells, T cells, and APCs, and CD40L-blockade reduced disease in numerous animal models of autoimmunity (181). Tolerogenic vaccination with FVIII and anti-CD40 prevented subsequent development of anti-FVIII antibodies during rechallenge (182) (**Table 3**).

Other approaches include genetic modification of co-stimulatory signals. Antigen delivered with a CRISPR-Cas9 plasmid and guide RNAs toward CD80, CD86, and CD40 disrupted co-stimulation by DCs, reduced inflammatory cytokines, increased Tregs, and suppressed disease in a model of T1D (183) (**Table 5**). Administration of a DNA vector encoding membrane-bound autoantigen together with a B7.1/CD40L mutant fusion protein binding to CTLA-4 but not CD28, providing co-inhibitory but not co-stimulatory signals, reduced disease incidence in murine model of T1D (184) (**Table 3**).

Intercellular adhesion molecule 1 (ICAM-1) is a cell surface glycoprotein most known for its role in leukocyte migration. Inhibition of ICAM-1 can block T cell activation and induce tolerance by disrupting T cell-APC interactions, inhibiting co-stimulation, promoting PD-L1 expression, and by inducing T cell anergy or exhaustion (185). A tolerogenic vaccine comprised of hyaluronic acid with autoantigen and an ICAM-1 inhibitory peptide suppressed disease in EAE (186–188). In another tolerogenic vaccine, fusion molecules of antigen and ICAM-1 inhibitory peptide prevented the development of T1D (189) and suppressed EAE (190) (**Table 3**).

OX40 is a TNF receptor superfamily member expressed by activated T cells and resting Tregs, which acts as a co-stimulatory molecule promoting cell proliferation (191). Tolerogenic vaccination of prediabetic mice with antigen and an OX40 agonistic antibody reduced diabetes incidence and increased antigen specific Tregs (192) (**Table 3**).

#### 2.4.3 Activation of inhibitory receptors

Programmed cell death protein 1 (PD-1) is an immune checkpoint molecule that induces T cell apoptosis and suppresses conventional T cell activation and favors development of Tregs (193). Treatment with tolDCs engineered to express PD-L1 and MOG reduced antigen-specific T cell responses and suppressed MOG induced EAE but not MBP induced EAE (148, 149) (**Table 3**).

B and T lymphocyte attenuator (BTLA) is an inhibitory receptor structurally related to PD-1, activation of which leads to the suppression of T cell activation (194), and BTLA-expressing DCs promoted Treg development (195). Adoptive transfer of bone marrow derived DCs treated with a nanoparticle containing antigen and a BTLA-encoding plasmid suppressed EAE (196) (**Table 3**).

Fas is a death receptor inducing apoptosis upon binding to Fas ligand (FasL). A tolerogenic vaccine using FasL-conjugated microparticles containing monocyte chemotactic protein-1 (MCP-1) recruited T cells and then induced their apoptosis. When coupled with respective antigens, the microparticles could suppress EAE and prevented development of T1D in pre-diabetic mice (197). (**Table 5**) A tolerogenic vaccine aiming for engagement of multiple inhibitory receptors for immune suppression used microparticles displaying surface PD-L1-Fc, anti-Fas, and self-marker CD47, and containing TGF-β. The vaccine led to reduced T cell infiltration and EAE suppression (198, 199) (**Table 5**).

CD22 and siglec G are inhibitory receptors inhibiting B cell receptor (BCR) signaling, thus suppressing B cell responses (200). Tolerogenic vaccination with liposomes displaying antigen and CD22/siglec G ligands induced antigen-specific tolerance in mice and reduce development of anti-drug antibodies toward FVIII in a model of hemophilia A (201) and reduced antigen-specific antibody production (202) (**Table 3**). Encapsulation of rapamycin in CD22L autoantigen liposomes suppressed arthritis in mice (203–205) (**Table 5**).

### 2.5 Other adjuvants

#### 2.5.1 Modulators of apoptotic pathway signaling

Apoptotic cells are cleared by phagocytic cells via anti-inflammatory mechanisms, which in part is mediated by phosphatidylserine exposed on the apoptotic cell surface (206). Therefore, phosphatidylserine liposomes, O-phospho-L-serine (OPLS), or pro-apoptotic factors have been used as tolerogenic adjuvants to reduce antigen immunogenicity.

In hemophilia A, both co-delivery of FVIII with OPLS and tolerogenic nanoparticle vaccination with FVIII encapsulated in phosphatidylserine liposomes led to a reduction in anti-drug antibodies toward FVIII in mice (207, 208), and antigen-containing phosphatidylserine liposomes reduced disease incidence in a model of T1D (209). In addition, phosphatidylserine liposomes loaded with collagen peptide and the immunomodulator leflunomide, which inhibits the mitochondrial enzyme dihydroorotate dehydrogenase preventing uridine synthesis, suppressed CIA in mice (210). Furthermore, a DNA vaccine encoding antigen and the pro-apoptotic protein BAX, promoting apoptosis in cells expressing antigen, suppressed T1D via modulation of APC function and promotion of Treg development (211, 212) (**Table 4**).

#### 2.5.2 TLR agonists

TLR agonists are well known inflammatory stimuli and often used as adjuvants in immunogenic vaccines to enhance the immune response to the target antigen (213). However, signaling through microbial pattern recognizing receptors might also protect from development of autoimmunity. Dose and administration of the TLR agonist affects if the response is immunogenic or tolerogenic, it is believed that a short-term, high-dose stimulation will result in immunogenic responses whereas low-dose, repeated stimulation results in tolerance (214).

TLR4 agonist LPS is often used in cultures of DCs to induce maturation and enhance their antigen presenting capacity. Antigen-loaded LPS-treated DCs suppressed EAE while non-treated DCs did not (215). A recombinant fusion protein of autoantigen and flagellin A, TLR5 agonist, induced production of IL-6 and IL-10 in DCs and reduced T cell-driven inflammation in a murine model of intestinal allergy (216, 217). Co-delivery of flagellin B and antigen reduced disease in models of allergy (218, 219) and a fusion protein of antigen and flagellin B reduced disease and IgE responses in allergy (220) (**Table 4**).

TLR9 agonist CpG DNA has been used in different tolerogenic vaccines in combination with other adjuvants. Treatment with a hydrogel vaccine containing CpG DNA, antigen, and GM-CSF prevented and delayed disease onset in pre-diabetic mice, and the inclusion of CpG DNA enhanced efficacy compared to GM-CSF alone (131). Tolerogenic vaccination with antigen, CpG DNA, and heat shock protein 60 induced an antigen-specific increase in IL-10 production and reduced disease severity in a model of arthritis (221) (**Table 5**).

#### 2.5.3 Glycans and glycan-binding proteins

The use of glycans in tolerogenic vaccine design can mediate tolerance by targeting antigen to APCs expressing receptors for these glycans, promoting its uptake and processing, while concurrently inducing immunoregulatory effects in DCs (14, 222, 223).

β-glucan is a polysaccharide naturally occurring in cell walls of plants, bacteria, and fungi, and binds to Dectin-1 on myeloid cells. In a model of T1D, treatment with β-glucan and antigen led to increased protection from disease compared to treatment with β-glucan or antigen alone, and promoted tolDC features and increased Tregs (224). Conjugates of allergens to mannan, targeted these antigens to APCs expressing mannose and C-type lectin receptors, and promoted a tolerogenic response in comparison to native allergens *in vitro* and *in vivo* (222, 223). Skin-prick tests with mannan-conjugated grass pollen allergoids caused less inflammation than native allergens in patients with grass pollen allergy (225), and immunization of mice with mannan-allergoid conjugates led to tolerogenic responses and increase in Foxp3^+^ Tregs compared to native antigen (225, 226) (**Table 4**). Mannan-allergoid conjugates have been tested for dust mite and grass pollen allergens in two phase II clinical trials, showing improvement in nasal provocation test (227, 228) (**Table 6**).

Galectin-1 is a glycan-binding protein with diverse functions, including modulation of DCs and T cell responses (229). In a study of EAE, treatment of DCs with galectin-1 led to tolDC differentiation that suppressed EAE when loaded with relevant autoantigen (230). The disease suppression was dependent on IL-27 and IL-10 induced by galectin-1 (230) (**Table 4**).

## 3 Discussion

Tolerogenic vaccines are promising experimental treatments for a wide range of conditions, including autoimmune disease, anti-drug antibody responses, transplantation rejection, and hypersensitivity (3, 6). Successful reintroduction of immune tolerance via tolerogenic vaccination would have numerous benefits over traditional immunosuppression or immune modulation. First, tolerance could be durable as tolerogenic vaccines may deplete or inactivate pathogenic cells, while concurrently inducing long lived suppressive Tregs and/or regulatory B cells which can self-renew and persist (231, 232). Second, tolerogenic vaccines engaging antigen-specific Treg responses may engender bystander suppression and infectious tolerance to suppress autoimmune responses to unknown antigens (3, 233). Third, tolerogenic vaccines may have efficacy with minimized toxicity as they modulate the antigen-specific response, leaving the rest of the immune system intact. Together these characteristics could constitute a functional cure.

Although tolerogenic adjuvants are not always necessary in tolerogenic vaccines (3, 234, 235) addition of tolerogenic adjuvants have the potential to greatly enhance the efficacy of tolerogenic vaccines by several mechanisms. Immunosuppressive or anti-inflammatory tolerogenic adjuvants promote an anti-inflammatory environment upon antigen encounter, thereby reducing the risk of unwanted inflammatory responses, anaphylaxis or disease exacerbation when re-introducing disease-relevant antigens. Immunomodulatory tolerogenic adjuvants can steer the antigen-specific immune response in desired direction, and cell-targeting adjuvants ensure vaccine delivery to intended cell types and minimizes off-target effects. Additionally, tolDC transfer is a common tolerogenic vaccine modality, but this type of tolerogenic vaccine is associated with high costs and difficulties of standardization across patients. Therefore, tolerogenic vaccines using adjuvants to deliver antigen to and modulate DCs *in vivo* may represent a more feasible treatment option.

A major limitation to the development of tolerogenic vaccines is a lack of understanding of the autoantigen pools that drive autoimmune diseases. Few autoimmune diseases have limited and well-defined antigen pools, while most autoimmune disease have numerous, poorly defined or undefined antigen pool, and disease antigens may change through time or differ across patients. Therefore, initial clinical trials using tolerogenic vaccines have focused on conditions which have relatively defined antigen pools such as celiac disease, pemphigus vulgaris, T1D and anti-drug antibody responses. However, preclinical data suggest that induction of tissue-specific Tregs may circumvent the need-to-know exact antigens involved in disease as these vaccine-induced tissue-specific Tregs can traffic to the inflamed tissue and exert suppressive functions via bystander suppression or infectious tolerance to suppress immune responses to unknown antigens involved autoimmune disease (3, 233). Utilizing adjuvants to expand or enhance Treg responses, may therefore enable further application of tolerogenic vaccines also in autoimmune diseases with complex autoantigen pools.

Another unknown is if tolerogenic vaccines that induce tolDCs and/or Tregs can suppress preexisting pathogenic B cell that were licensed by CD4 T cell and have limited ongoing interactions with either Tregs or TolDCs. Therefore, tolerogenic vaccines might have to address B cells separately. This could be achieved by B cell targeting or B cell suppression, such as by adjuvants signaling via Siglec G and CD22 or using adjuvants with effects on both B and T cell responses. This approach could be combined with a tolerogenic vaccine design acting on tolDC and/or T cells to prevent further activation of novel pathogenic B cell clones.

In conclusion, tolerogenic vaccines may be the therapeutics of the future for autoimmune and inflammatory conditions. Tolerogenic adjuvants are powerful tools with capacity to both enhance antigen-specific tolerance as well as reduce the risk of unwanted inflammatory responses or off-target effects.
